# Anomalous Dispersion via Dissipative Coupling in a Quantum Well Exciton-Polariton Microcavity

**DOI:** 10.1021/acs.nanolett.6c00554

**Published:** 2026-05-22

**Authors:** Dąbrówka Biegańska, Maciej Pieczarka, Christian Schneider, Sven Höfling, Sebastian Klembt, Marcin Syperek

**Affiliations:** † Department of Experimental Physics, Faculty of Fundamental Problems of Technology, 49567 Wrocław University of Science and Technology, Wybrzeże Wyspiańskiego 27, 50-370 Wrocław, Poland; ‡ Carl von Ossietzky Universität Oldenburg, Fakultät V, Institut für Physik, 26129 Oldenburg, Germany; § 9190 Julius-Maximilians-Universität Würzburg, Physikalisches Institut and Würzburg-Dresden Cluster of Excellence ctd.qmat, Lehrstuhl für Technische Physik, Am Hubland, 97074 Würzburg, Germany

**Keywords:** exciton-polariton, dissipative coupling, anomalous dispersion, non-Hermitian systems, AlGaAs polaritons

## Abstract

Although energy level repulsion is typically observed in interacting quantum systems, non-Hermitian physics predicts the effect of level attraction, which occurs when significant energy dissipation is present. Here, we show a manifestation of dissipative coupling in a high-quality AlGaAs-based polariton microcavity, where two polariton branches attract, resulting in an anomalous, inverted dispersion of the lower branch in momentum dispersion. The dissipative coupling is explained by the interaction with an indirect exciton, acting as a highly dissipative channel in our system. Using angle-resolved photoluminescence measurements we observe the evolution of the level attraction with exciton-photon detuning, leading to changes in anomalous dispersion shape within a single sample, and the observed dispersions are well captured within a phenomenological model. Our results present a new mechanism of dissipative coupling in light-matter systems and offer a tunable and well-controlled AlGaAs-based platform for engineering the non-Hermitian and negative mass effects in polariton systems.

## Graphical abstract

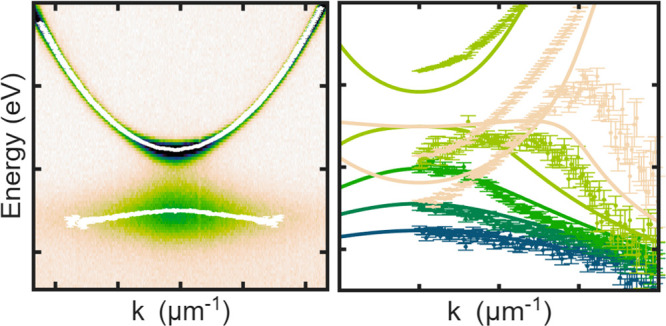

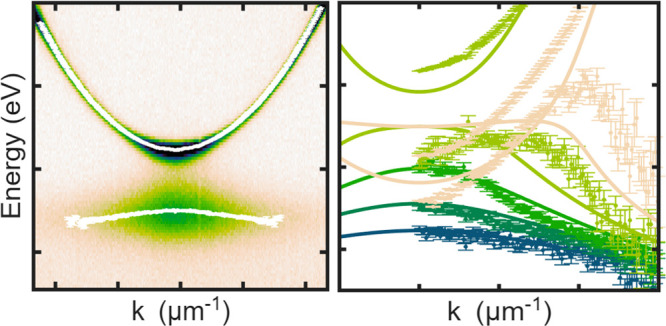

In interacting quantum systems, it is typical to observe level repulsion. When two modes couple and intermix, the resulting energy levels anticross, avoiding degeneracy at resonance. If the strongly interacting states are photons and excitons confined in planar microcavities, the resulting eigenstates appear as two exciton-polariton branches, schematically depicted in Figure 1(a). Lower polaritons are characterized by a nearly parabolic dispersion at small wavevectors, with a small and positive effective mass, inherited largely from the photonic component. The mode dispersion and particle effective mass can be further engineered, yet it requires additional sample processing or sophisticated excitation schemes.^2–5^

**Figure 1:**
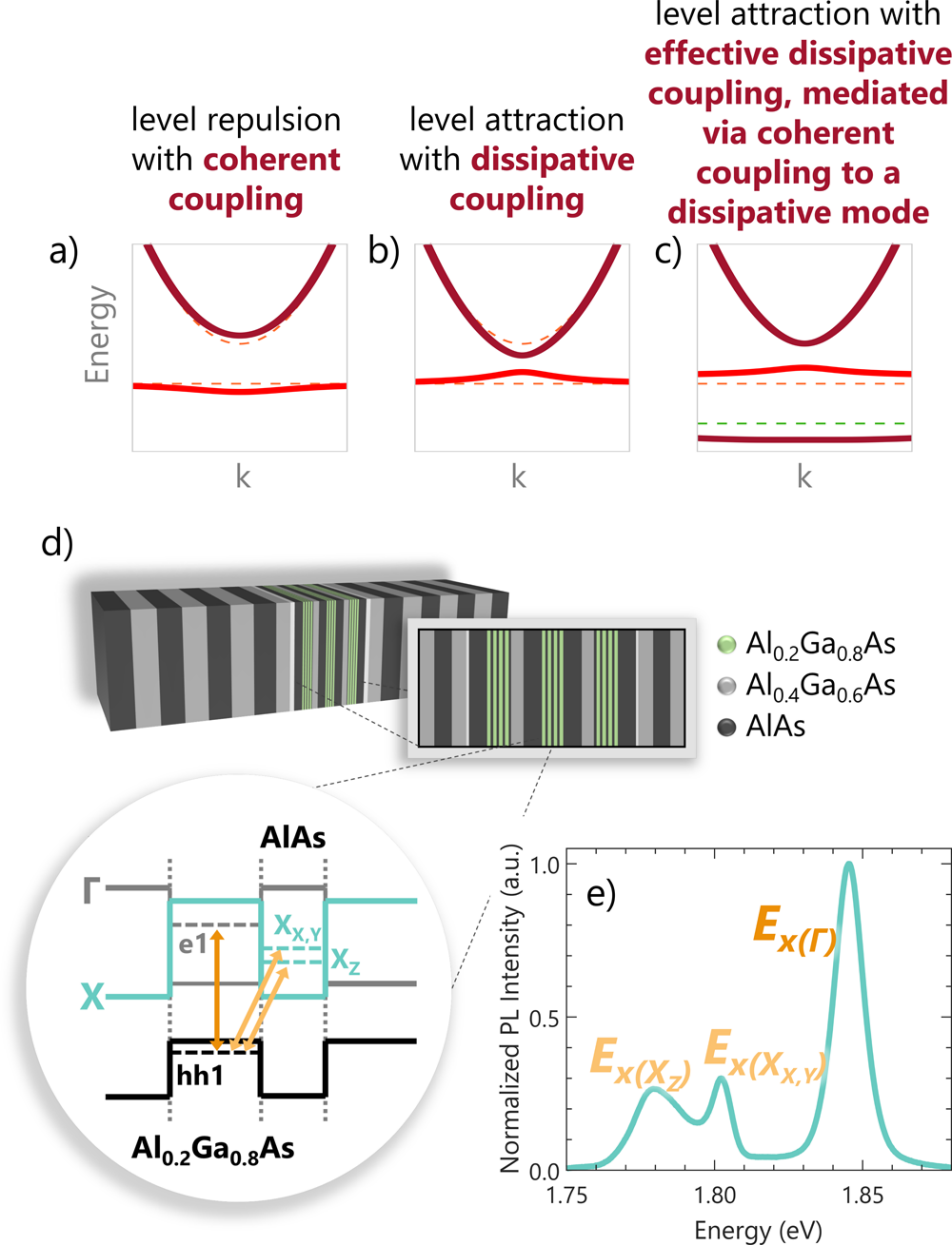
(a) Levels of a strongly coupled system of two coherently coupled modes, showing level repulsion. (b) Level attraction of the same two modes, coupled with an imaginary coupling. (c) Schematic visualization of a similar level attraction effect, but coming from a real coupling between three modes, one of which is strongly dissipative. (d) Schematics of the investigated microcavity, with a close-up of the active layer. In the system band structure solid lines show the edges of the X, Γ and valence bands of one period of the repeated layers. Dashed lines indicate the quantized electron (*e*1, *X*_*X*,*Y*_, *X*_*Z*_) and heavy hole (*hh*1) levels in two adjacent layers. Carriers occupying these levels subsequently form three excitons present within the system (indicated with orange arrows), when subject to Coulomb interactions. (e) The photoluminescence spectrum of the bare quantum well system, with the top Bragg reflector etched away. The three observed lines correspond to the three excitons: direct Γ exciton (*E*_*x*(Γ)_), and two indirect excitons: *E*_*x*(*X*_*X*,*Y*_)_ and *E*_*x*(*X*_*Z*_)_. Measured photoluminescence linewidths are 8.3, 11.3, and 20 meV respectively, influenced by the valley mixing, interface inhomogeneities and sample disorder, as discussed in ref.^1^

However, in all open systems, losses are inevitable, and interactions and eigenstates are strongly affected by dissipation. When dissipation becomes equally important to the coherent coupling, the emergent states can attract (instead of repelling), even without additional potential, as it is schematically visualized in Figure 1(b). The attraction effect is analogous to the classical in-phase oscillations of dissipatively coupled pendulums.^6^ When the dissipative coupling affects two quantum mechanical oscillators with parabolic dispersions, the resulting band has a negative curvature parabolic wavevector dependence, directly representing the negative effective mass of the emergent state.

In light-matter systems the influence of dissipative coupling has been experimentally observed in photonic-crystal cavities containing single quantum dots.^7^ In two-dimensional polaritonic systems, however, while there were some first experimental hints in rather low-Q microcavities containing monolayer semiconductors,^8,9^ clear studies in narrow-linewidth systems have been elusive so far. The level attraction phenomenon has been studied mainly in other contexts, such as magnons,^6,10,11^ microwave cavities^12^ or mechanical systems.^13,14^ Dissipative coupling has been suggested as a potential mechanism for the creation of entangled states, as a new tool in the design of superconducting qubits,^10,11^ for the development of metamaterials,^10^ but also as a mechanism beneficial in cavity spintronics.^11^

Even though the level attraction and the anomalous dispersion can be phenomenologically described by the imaginary coupling between the two states, the physical origin of the effect is not always clear and varies between systems. Interestingly, it has been shown how, in a physical system, the dissipative type of coupling can be realized by coupling two oscillators reactively to a third, highly dissipative entity,^15^ as schematically depicted in Figure 1(c). The third-party mode in cavity systems can come from an invisible cavity mode with extremely high leakage or dissipation. This mechanism has been successfully used to explain the level attraction in magnon cavities,^15^ yet it has never been considered in an exciton-polariton context.

Regardless of the mechanism, the negative mass of such an inverted state can be used in a wide range of studies on non-Hermitian effects or topology.^16–25^ It manifests itself in the particle’s dynamics, so that its group velocity and momentum have opposing directions.^9,26^ In addition to substantial fundamental interest, this, in turn, can be employed to control wavepacket dynamics,^26^ hydrodynamics,^27^ or cause resonance trapping.^28^

In this work, we unequivocally demonstrate the level attraction manifested as an inverted anomalous dispersion in the AlGaAs exciton-polariton system. We investigate the mechanism of dissipation in our structure, crucial for the attraction to occur. In contrast to previous studies, our III–V semiconductor sample not only hosts conventionally studied Γ-excitons in the quantum wells (QWs), coherently coupled to photons, but also lower-energy spatially- and momentum-indirect X-excitons, which are strongly prone to dissipation. We show that the source of dissipation in our structure is the lower-energy indirect state, acting as a draining channel for both photons and electrons. This highly dissipative mode allows for the dissipative coupling to become sufficiently strong to surpass the coherent exciton-photon coupling, and result in inverted eigenstate dispersion. Finally, we demonstrate the superiority of our material system in comparison to previous realizations, owing to its high tunability, ease of design, and huge potential for non-Hermitian phases engineering, by showing a change of the dispersion shape as a function of exciton-photon detuning.

We studied an AlGaAs/AlAs optical microcavity, designed for room-temperature polaritonics.^29^ However, in this work, we focus on experimental observations made at cryogenic temperature of 4 K, benefiting from the narrow polaritonic linewidths. The sample consists of twelve 9 nm-wide Al_0.20_Ga_0.80_As QWs, separated by 4 nm AlAs barriers and placed in a λ/2-AlAs cavity, surrounded by AlAs/Al_0.40_Ga_0.60_As distributed Bragg reflectors (DBRs). The sample schematic is depicted in Figure 1(d) and a detailed description of the sample composition can be found in the Supporting Information, section I.

Due to the high aluminum content affecting the band alignment, the structure hosts direct and indirect excitons in the QW.^1,29,30^ Apart from the conventional direct excitons composed of Γ-valley electrons and heavy holes confined in the QW layer, the structure also hosts lower-energy spatially and momentum indirect X-excitons.^1^ Since the order of X and the Γ-valley energy minimum in the conduction band is reversed for the Al_0.2_Ga_0.8_As QW and for the AlAs barrier material, the fundamental QW electron state resides in the barrier. This allows the formation of indirect excitons composed of X-valley electrons in the barrier Coulomb-correlated with Γ-valley heavy holes confined in the QW layer. Two lowest-energy optically active states relate to excitons consisting of X-valley electrons with different effective masses (longitudinal and transverse with respect to the spatial quantisation axis), forming *X*_*Z*_ and *X*_*X*,*Y*_ states, respectively. The single-particle energy levels are visualized in the QW band structure in Figure 1(d), using dashed lines. Measured spectrum of the bare QW active material is presented in Figure 1(e), where all excitonic transitions are indicated. The indirect nature of these excitonic states has been investigated in detail in our previous work.^1^

When embedded in a monolithic optical microcavity close to resonance with the Γ-state, direct excitons couple strongly to light, forming exciton-polariton quasiparticles.^29^ These states are characterized by the normal-mode splitting and present typical polariton dispersions (as shown in the Supporting Information, section II). However, herein we study the structure at very large negative Γ-exciton-photon detunings, Δ_Γ_ = *E*_*c*_ – *E*_*x*(Γ)_ < 0 (where *E*_*c*_ is the cavity mode energy and *E*_*x*(Γ)_ is the energy of the direct exciton in the QW). In this regime, the light-matter interactions are dominated by the coupling of the cavity optical mode to the indirect X-valley excitons, and the resulting states strongly differ from the typical exciton-polaritons under coherent light-matter coupling. The detuning is sufficiently large that the coherent coupling to the Γ-excitons becomes irrelevant. For convenience, throughout the rest of the paper, we will refer to the detuning as defined with respect to the higher-energy X-exciton, Δ_*X*_ = *E*_*c*_ – *E*_*x*(*X*_*X*,*Y*_)_.

To study the coupling between photons and X-excitons, we measured angle-resolved photoluminescence spectra in a wide detuning range, close to resonance with the X-excitons. When the photonic mode gets sufficiently close to the energy of the *X*_*X*,*Y*_ excitonic resonance, a new lower-energy state brightens up, with the dispersion curved in a distinctly inverted manner. An experimental example of such a momentum dispersion is presented in Figure 2(a), together with the peak energies of the two branches, extracted with a fitting procedure (see the Supporting Information, section III). An apparent and monotonous redshift of this mode’s energy with increasing wavevector can be seen in Figure 2(b), a dependence opposite to the higher energy photonic-like state. The two levels attract, causing the mirroring of their wavevector energy dispersions, mimicking the dispersion sketched in Figure 1(b). The negative curvature of such an inverted parabolic dispersion is directly linked to the negative effective mass of the lower mode - a rare phenomenon in exciton-polariton systems.^8,9,31,32^

**Figure 2:**
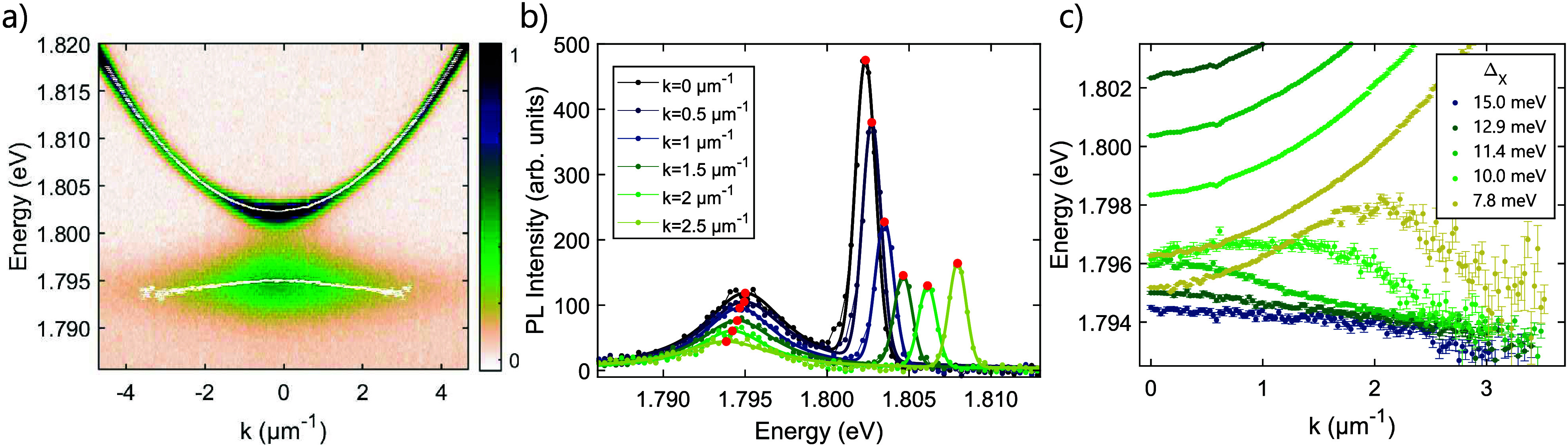
(a) Momentum-resolved photoluminescence image at a chosen exciton-photon detuning (linear color scale). Spectra crossections taken at several wavevectors are presented in (b) (connected dots), together with fitted curves (solid lines). Red dots show the energies of the two deconvoluted modes, extracted from fitting, also marked in white in (a). (c) Extracted mode dispersions at several exciton-photon detunings Δ_*X*_. Error bars indicate the fitting standard error. All spectra were obtained using low excitation power and using the same experimental conditions for each detuning.

Taking advantage of the cavity energy gradient (due to the thickness variation across the sample), we probed the negative mass states in a range of sample positions (detunings). As presented in Figure 2(c), decreasing the detuning between the cavity mode and the *X*_*X*,*Y*_-exciton energy Δ_*X*_ leads to an increase in attraction effect, with the anomalous shape of the lower branch becoming steeper and more distinct. Figure 2(c) shows the energies of two polaritonic branches extracted from fitting the photoluminescence measurements taken at different sample positions. Interestingly, around the positive photon to *X*_*X*,*Y*_-exciton detuning of approximately 10 meV the curvature changes from the inverted parabola-like with one energy maximum at *k* = 0 to an anomalous shape with two distinct and symmetric maxima at *k*≠0. Similar dispersion shapes have been observed before in different structures in both regimes,^8,9^ yet never in the same material system, nor in a single sample. The corresponding change of the effective mass value with detuning is presented in the Supporting Information, section VII.

At negative detunings Δ_*X*_, only one branch appears in the photoluminescence spectrum, with the standard parabolic shape of the dispersion resembling the one of a photonic mode, as presented in the Supporting Information, section II. For further discussions, we focus solely on the level attraction region. We also note that the anomalous dispersion is not visible in the reflection spectra (see Supplementary Discussion, section VIII).

To understand the source of level attraction, we have to recall the existence of the *X*_*Z*_-electron exciton, with energy below both the *X*_*X*,*Y*_ exciton and the photonic mode, which inclusion proves to be crucial in the theoretical description of the data. To describe our system and quantify the mechanism of level attraction, we used a general three coupled oscillator model, predicting attractive level crossing via the existence of a dissipative mode.^15^ Even though the level attraction has previously been described with the use of imaginary coupling between two oscillators,^9,33^ in^15^ the authors show how, in a physical system, the dissipative coupling can be realized by coupling two oscillators to a third highly dissipative one, even if the mode is invisible. In our case, the third-party mode could be identified as the *X*_*Z*_ -exciton.

The model can be represented by a 3 × 3 non-Hermitian matrix:

$$H=\left(\begin{array}{lll} E_{1} & V & g_{1}\\ V & E_{2} & g_{2}\\ g_{1} & g_{2} & E_{0} \end{array}\right)=\left(\begin{array}{lll} E_{c}-i\gamma_{c} & V & g_{1}\\ V & E_{x\left(X_{X,Y}\right)}-i\gamma_{x} & g_{2}\\ g_{1} & g_{2} & E_{x\left(X_{Z}\right)}-i\gamma_{0} \end{array}\right)$$ 1

In this approach, two oscillators with intrinsic decay (with energies of *E*_1_ and *E*_2_) are coupled to each other coherently via *V*, and to the third oscillator *E*_0_, which is strongly damped, γ_0_ ≫ γ_*c*_, γ_*x*_. Significant dissipation of the third state is crucial for the level attraction and for *E*_0_’s strong influence on the *E*_1_ and *E*_2_ dispersions, when the real coupling terms *g*_1_ and *g*_2_ are sufficiently large to surpass the coherent coupling *V*. In such conditions, these terms can effectively act as complex coupling between the two modes,^9,10,15^ provided that *E*_1_ and *E*_2_ are nearly resonant, which is further discussed in the Supporting Information, Section IV. In a regime of high coherent coupling between the two resonances and a weak dissipation of the third mode, all eigenstates repel, as is typically observed in exciton-polariton systems.^34–38^ The 3 × 3 matrix can be regarded as a more general counterpart of the non-Hermitian 2 × 2 model with imaginary coupling between two oscillators used before,^9,33^ describing a broader case of dissipative coupling leading to the attraction effect (for the detailed discussion, see the Supporting Information, Section IV).

We schematically visualize the model and the involved oscillators in Figure 3(a). In our structure, two coupled resonances are the photonic mode *C* and the *X*_*X*,*Y*_ exciton, with energies and decay rates of *E*_*c*_, *E*_*x*(*X*_*X*,*Y*_)_ and γ_*c*_, γ_*x*_, respectively. The lower-energy *X*_*Z*_ excitonic resonance acts as a dissipative mode and is characterized by the energy of *E*_*x*(*X*_*Z*_)_ and the dissipation γ_0_. The coupling between photons and *X*_*X*,*Y*_ excitons inside the microcavity (*V*) is expected to be weak, due to the space- and momentum- indirect nature of the excitonic resonance. In contrast, *X*_*Z*_-exciton is expected to couple to light more efficiently, as the spatial symmetry breaking allows for its recombination without the assistance of phonons, due to the weakening of the momentum-conservation rules, regardless of its indirect nature.^1,39,40^ The coupling between the two indirect excitons is enabled via transfer of electrons between states and transitions from the higher *X*_*X*,*Y*_ to the lower *X*_*Z*_ electronic state, as evidenced by complex temporal dynamics^1^ and previous studies.^41–44^ Both couplings *g*_1_ and *g*_2_ are therefore expected to play a significant role in the system, with the *g*_1_ value expected to be much larger than *V*. The energies of both excitonic resonances can be directly inferred from the photoluminescence measurements of the bare QW structure (see Figure 1(e) and ref 1).

**Figure 3:**
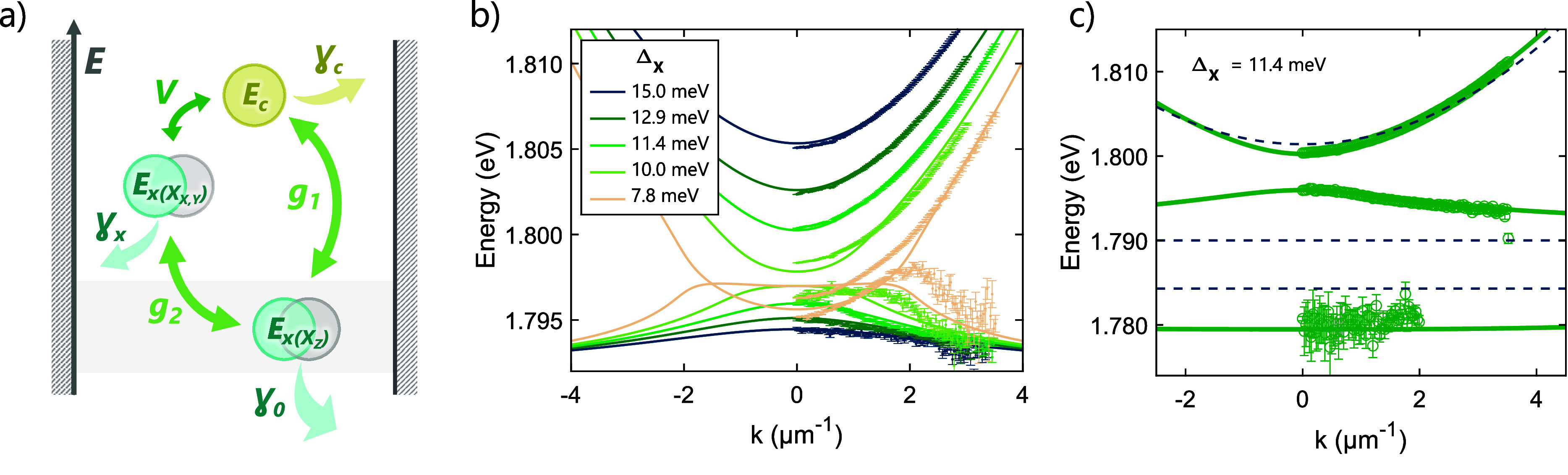
(a) Schematic visualization of the three coupled oscillators model and its application in our system. The coupled particles (photon (*E*_*c*_) and two indirect excitons (*E*_*x*(*X*_*X*,*Y*_)_ and *E*_*x*(*X*_*Z*_)_)) are shown on a schematic energy scale, with their intrinsic decays sketched as broad arrows, while the couplings are presented as two-sided arrows. (b) Comparison of the model lines with experimental level branches at several exciton-photon detunings, plotted in corresponding colors. Model parameters are described in the main text. (c) Example dispersion at a single photon-*X*_*X*,*Y*_-exciton detuning of 11.4 meV. Dashed lines mark the dispersions of a bare photonic mode and two indirect excitons, open points are the fitted peak positions of the three polaritonic branches, and solid green lines are the model dispersions. For clarity, the experimental data is only presented for positive k.

Using this approach, we modeled our experimental dispersions, as presented in Figure 3(b). Experimental points are the extracted peak energies of the two polaritonic branches at several exciton-photon detunings Δ_*X*_, and solid lines show the fitted model eigenstates. Additionally, in Figure 3(c) we show all three of the model eigenstates at the exciton-photon detuning of 11.4 meV, as well as the dispersions of a bare photonic mode and two indirect excitons (dashed lines). We note that in most measurements the lowest-energy mode cannot be seen in the photoluminescence spectra, except near the Δ_*X*_ ≈ 11 meV detuning, hence we used only two states in the dispersion modeling.

The model results show very good correspondence with the measured dispersions. The model well reflects the anomalous shape of the lower branch dispersion and captures a clear transition between its monotonic (with a single maximum at *k* = 0) and nonmonotonic (with maxima at finite wavevectors) |*k*|-dependence when decreasing Δ_*X*_. At larger detunings, the model dispersions match experimental points nearly perfectly, demonstrating the change in curvature around *k* = 0, linked to the dissipative level attraction. Discrepancies between the model and the experimental curves become visible only at smaller positive exciton-photon detunings (Δ_*X*_ ≤ 10 meV). This may arise from the fact that to model our data we set all the parameters constant throughout this detuning range (apart from the photonic mode energy), which is a simplified approach. All three decay constants, as well as the level energies, can vary across the sample, due to the local disorder and the layer width change, however such change is impossible to capture in our structure and would significantly add to the complexity of the fit. Nevertheless, the model describes our system very well in a large range of exciton-photon detunings, even when using only one set of parameters. Moreover, a high agreement between the model line and the third state detectable at the detuning Δ_*X*_ = 11.4 meV presented in Figure 3(c), despite not using this state in the fitting, further proves the applicability of our model.

The extracted exciton-photon couplings are *V* = 0.1 meV and *g*_1_ = 10.6 meV, while the coupling between two X-excitons *g*_2_ is 17 meV. As expected, the coherent coupling between the photonic mode and the spatially and momentum indirect *X*_*X*,*Y*_ exciton is much smaller than other energies in our system. The highly dissipative *X*_*Z*_ state couples to light more efficiently, what is likely a result of the symmetry breaking effect described above. The most influential interaction comes from the nonradiative coupling between the two X-excitons. The extracted decay rates of all states are γ_*c*_ = 0.1 meV, γ_*x*_ = 0.01 meV and γ_0_ = 41 meV. The model photon linewidth value corresponds to a lifetime of approximately ∼ 6 *ps*, which is a value expected for this microcavity, subject to disorder and operating at large detuning from the designed wavelength.^45^ A small line broadening of the *X*_*X*,*Y*_ state originates from its longer lifetime, expected from its indirect nature. On the other hand, the large broadening γ_0_ of the *X*_*Z*_ exciton points to its dissipative role and it is crucial to obtain level attraction in our system. We note that the model value is larger than the measured photoluminescence linewidth broadening of this state of ∼20 meV, measured with the top mirror removed from the cavity,^1^ however, the observed emission linewidth cannot be directly translated into the homogeneous broadening. Photoluminescence broadening consists of both homogeneous and inhomogeneous parts, but, at the same time, it can be narrowed by a Purcell effect, resulting from the formation of a very low-Q-factor half-microcavity.^34^ The large damping of this mode likely comes from the sensitivity of these states to structure inhomogeneities, stemming from their ground state nature, and affecting their lifetime and transport properties, as discussed in detail in^1^ and shown before.^46^ Overall, the model accurately describes our system and reveals the highly damped *X*_*Z*_ excitons as the source of the level attraction and the inverted polariton dispersion. The importance of the damped mode inclusion is presented in section V of the Supporting Information.

In summary, we have observed the anomalous dispersion of the polaritonic branch in an AlGaAs-based microcavity, characterized by the negative effective mass. Our AlGaAs-based semiconductor system offers precise high-quality growth and design of the layers, fine-tuning its properties, which will uniquely allow to tailor the coherent coupling and the dissipation. We have shown how the presence of and the coupling to the indirect excitonic state energetically below the excitonic and photonic resonances, which acts as a channel of loss, can manifest itself as a dissipative coupling between these states. Furthermore, we have observed the evolution of the system eigenstates with varying detuning, showing the shift and the change of the eigenstate dispersion curvature. We show two regimes of anomalous dispersion shape, with eigenstate energy maxima at *k* = 0 and *k*≠0 in a single sample.

Importantly, we present the effect of level attraction in a new experimental platform, with a new source of the dissipative coupling in the exciton-polariton context. Previously, the anomalous dispersion of exciton polaritons in unstructured samples has been indicated solely in transition metal dichalcogenide-based samples,^8,9,32^ with supporting models applicable only for many-particle excitons in heavily doped samples with complex interactions,^8^ or for media with a strong influence of exciton–phonon interactions.^9^ Previously studied systems lacked the presence of a tunable and energetically lower state providing a channel of loss, which proves to be crucial in our structure. Even more importantly, they also lacked the excellent linewidths, making the dispersion shape less distinct and rendering interpretations of the observed dispersions less robust.

Apart from narrow linewidths, our system provides a great opportunity for level attraction tuning, via changing the detuning between the resonances, owing to the sample’s wedged growth, allowing access to different anomalous dispersion curvatures in a single platform. It enables effective mass engineering without the need of additional sample processing steps (such as patterning or etching) or complicated excitation schemes (e.g., structured beams). In our case, the mass can be engineered during the typical sample growth, with no further steps required, showing a clear path for future studies or device design.

Anomalous dispersion can be employed in novel studies of non-Hermitian effects,^16,17,47^ nontrivial dynamics and hydrodynamics^26,48^ and in studies of analogue systems.^21,49,50^ It allows access to a plethora of studies on exceptional points and related phenomena, such as winding of the complex eigenenergies, chiral modes, topological lasing, or enhanced perturbation, among others.^18,24,51,52^ The optical, easily experimentally accessible platform of high quality and high adjustability presented in this work makes our finding relevant and desirable far beyond the exciton-polariton context.

## Supplementary Material



## Notes

Discussion and details on the photoluminescence measurements at different detunings, photoluminescence fitting and dispersion extraction, discussion of the model transformation, nonhermiticity and coupling importance, calculated Hopfield coefficients, effective mass, and a note on reflectivity measurements (PDF)

## #

Monash University, Clayton, Victoria 3800, Australia
