# Supplementary material for: Anomalous Dispersion via Dissipative Coupling in a Quantum Well Exciton-Polariton Microcavity
Source: Nano Lett. 2026 May 22;26(22):7279–85. doi: 10.1021/acs.nanolett.6c00554 (PMC13309004; doi:10.1021/acs.nanolett.6c00554)
Supplement: Supplementary file 1 [file nl6c00554_si_001.pdf]

SUPPLEMENTAL MATERIAL
Anomalous dispersion via dissipative coupling in a quantum well exciton-polariton microcavity

D. Biegańska,[1,*] M. Pieczarka,[1] C. Schneider,[2] S. Höfling,[3] S. Klembt,[3] and M. Syperek[1]

[1] *Department of Experimental Physics, Faculty of Fundamental Problems of Technology, Wrocław University of Science and Technology, Wybrzeże Wyspiańskiego 27, 50-370 Wrocław, Poland*

[2] *Carl von Ossietzky Universität Oldenburg, Fakultät V, Institut für Physik, 26129 Oldenburg, Germany*

[3] *Julius-Maximilians-Universität Würzburg, Physikalisches Institut and Würzburg-Dresden Cluster of Excellence ct.qmat, Lehrstuhl für Technische Physik, Am Hubland, 97074 Würzburg, Germany*

I. SAMPLE AND EXPERIMENTAL DETAILS

The sample under study consists of twelve 9 nm-wide $Al_{0.20}Ga_{0.80}As$ QWs, separated by 4 nm AlAs barriers, distributed in three stacks of four (as visualised in Fig. 1(d) of the main text). The stacks are placed in a $\lambda/2$-AlAs cavity surrounded by $AlAs/Al_{0.40}Ga_{0.60}As$ distributed Bragg reflectors (DBRs), consisting of 28/24 mirror pairs in the bottom/top reflector, including 3 nm GaAs smoothing layers after each mirror pair in the local minimum of the electromagnetic field. The whole microcavity structure was grown by molecular beam epitaxy on the GaAs substrate. Lack of wafer rotation during growth results in a gradual change in the cavity length across the sample, allowing experimental access to a wide range of exciton-photon detunings. The photoluminescence spectrum of the bare quantum well system presented in Fig. 1(e) was taken on a different piece of the same sample, cleaved from the same wafer, however with the top Bragg reflector etched away [1].

To perform optical experiments, the sample was placed in the continuous flow liquid helium cryostat and cooled down to 4.2 K. It was excited by laser pulses from the OPO pumped by a Ti:Sapphire pulsed laser with 76 MHz repetition rate, generating a wavelength of around 620 nm. The beam was focused on a sample via a NA = 0.65 objective. The structure photoluminescence was then collected by the same objective and imaged on a slit of a monochromator (with a 1200 lines/mm groove density diffraction grating) equipped with a high-efficiency EMCCD camera. Imaging the Fourier plane by using four confocal lenses in the detection path allowed for the angle-resolved measurements.

II. PHOTOLUMINESCENCE MEASUREMENTS AT DIFFERENT DETUNINGS

Owing to a stopped rotation of the wafer during an active layer growth, resulting in a gradual change of the cavity length across the sample, we were able to access a very large range of exciton-photon detunings. We measured momentum-resolved photoluminescence spectra with the photonic mode scanning the energies of the whole cavity stopband.

As described in the main manuscript, when the photonic mode is sufficiently close to the direct Γ-electron exciton energy of 1.842 eV, one can observe the result strong coupling. Supplementary Figure 1(a) shows an example of far-field photoluminescence in this region, with the extracted energies of the lower polariton branch plotted as points. Additionally, lower polariton dispersions at several exciton-photon detunings ($\Delta_\Gamma = -18 \rightarrow 11$ meV) are presented in Supplementary Figure 1(b). One can see a clear change in the branch curvature, typical for the strong coupling regime in exciton-polariton studies. The extracted vacuum Rabi splitting at 4.2 K is $\hbar\Omega_\Gamma \approx 12$ meV.

When the energy of the photonic mode is lower, and gets closer to the indirect $X_{X,Y}$-exciton, we observe anomalous dispersion of one of the eigenstates, as described in the main manuscript and presented there in Figure 2. However, in the negative detuning range in this region, $\Delta_X < 0$, when the photon energy is lower than that of the $X_{X,Y}$-exciton, only one parabolic branch appears in the photoluminescence spectra. Two examples of such measurements are presented in Supplementary Figure 1(c) and (d). In the whole figure dotted lines mark the energies of three excitons present in the sample.

* dabrowka.bieganska@pwr.edu.pl

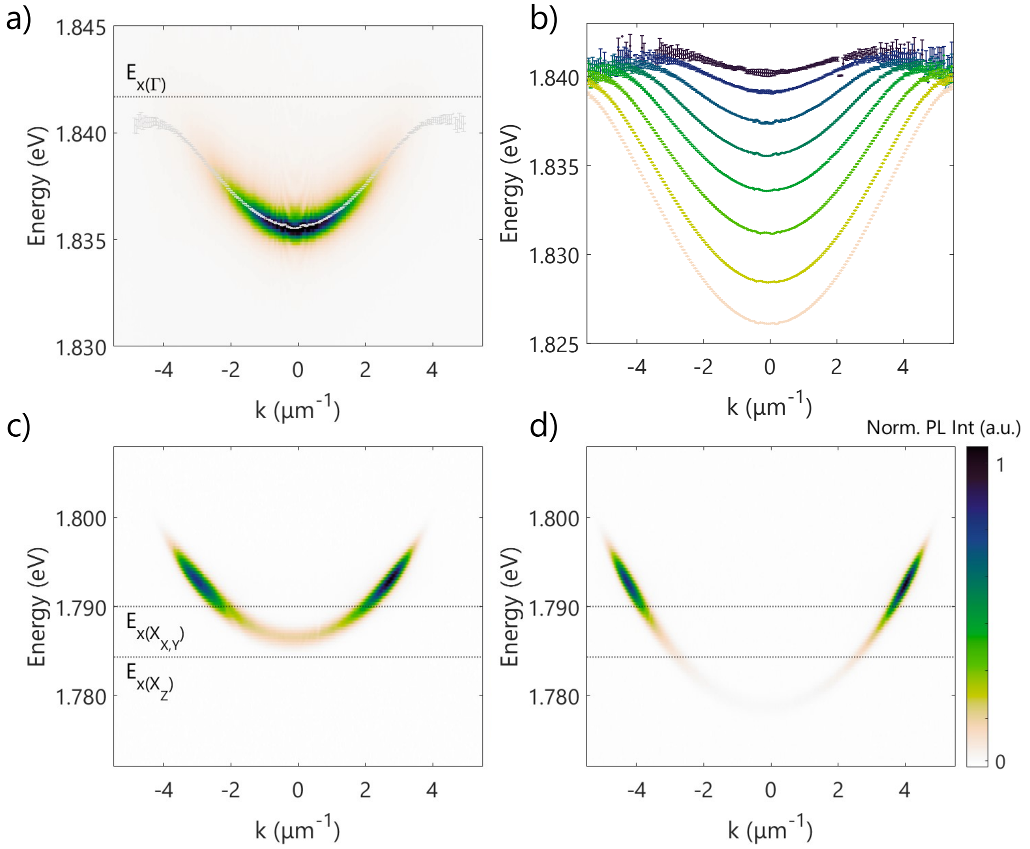

Supplementary Figure 1. (a) Momentum-resolved photoluminescence spectra in the strong coupling regime with the Γ-electron exciton, which energy is marked by a dashed line. Points mark the energy of the lower polariton branch, extracted by Lorentzian-curve fitting at each wavevector. (b) Dispersions of the lower polariton branch in the strong coupling regime in Δ_Γ (Γ-electron-exciton to photon) detuning range of around 11 (upmost curve) to -18 meV (lowest curve). (c), (d) Examples of photoluminescence spectra with photon energy below the $X_{X,Y}$- (c) and below both $X_{X,Y}$- and X_Z-electron-excitons (d).

III. PHOTOLUMINESCENCE FITTING AND DISPERSION EXTRACTION

To extract the energy dispersions of the three branches investigated in the main text, we fitted the photoluminescence spectra at each wavevector with a sum of a Lorentzian (lower central energy) and a Gaussian (higher energy) curves. An example of such a fitting is presented in the main manuscript in Figure 2(b). Additionally, two of the curves presented there are also shown in Supplementary Figure 2, together with the individual fitted profiles. Supplementary Figure 2(b) and (c) show the same curves but in logarithmic intensity scales, to increase the visibility of fit-to-data matching.

The choice of band profiles was carefully selected to obtain the highest agreement with the experimental data, while also minimizing the number of free fitting parameters. Voigt profiles are known to describe both the homogeneous and the inhomogeneous broadening of the experimental state lines, but their implementation adds significantly to the complexity of the fit. At selected wavevectors the double Voigt profile fitting was performed, giving peak energy results analogous to the Gaussian and Lorentzian sum fitting. However, the experimental data matching was poorer with the Voigt curves, visualized in lower goodness-of-fit metrics. At some momenta Voigt fit convergence was not achieved, due to the larger number of free fitting parameters in comparison to the Lorentzian and Gaussian curves. It is expected, that when the line shape is mostly Gaussian or Lorentzian and one of the broadenings is significantly higher than the other one, the Voigt model is over-parameterized and the convergence is not achieved. To better describe the experimental data and use consistent analysis for all wavevectors and all detunings, we choose the Gaussian and Lorentzian sum (with the fitted peak energy values used throughout the main manuscript). This combination of spectral profiles gave the best correspondence to the measured data, visualized in the highest coefficient of determination, R^2. Additionally, other choices of the spectral profiles, apart from the lower goodness-of-fit metrics, did not affect the extracted peak energies, with values equal within the standard error.

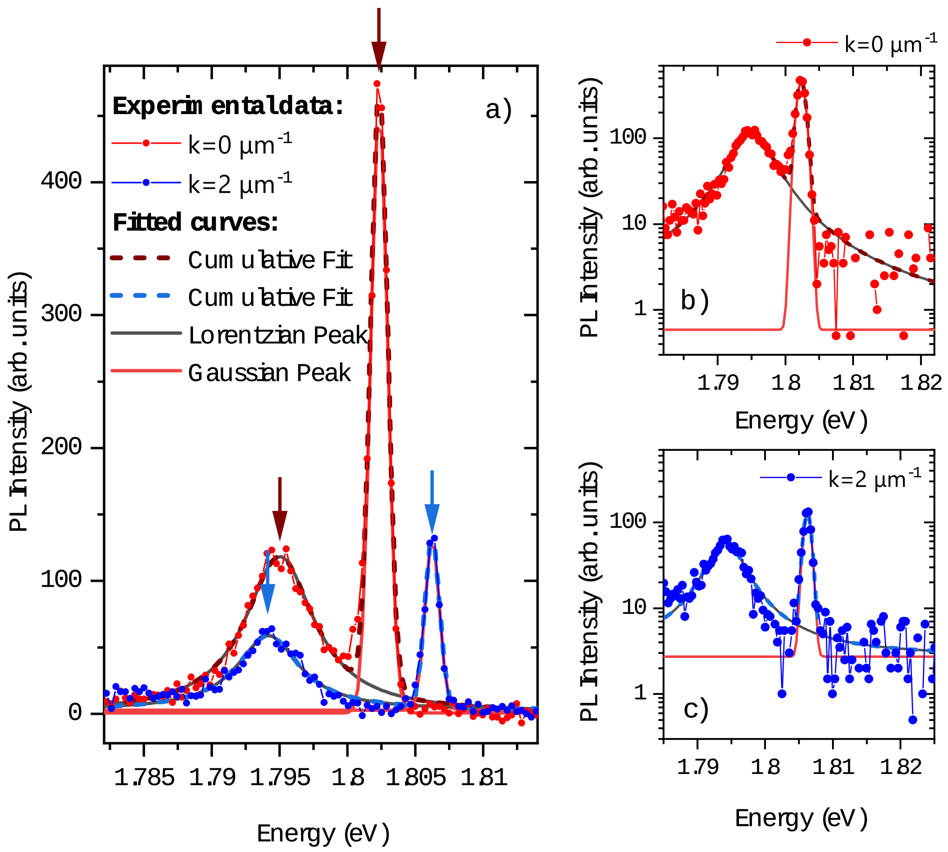

Supplementary Figure 2. Measured photoluminescence spectra taken at wavevectors $k = 0\ \mu m^{-1}$ and $2\ \mu m^{-1}$, at a detuning $\Delta_X = 11.4\ meV$, together with the fitted curves. Solid grey and red lines show the individual peaks of the convolution, while the dashed curves correspond to the final fitted model. Arrows in (a) indicate the peak central energies, extracted from the fitting, of the two peaks (shown also as red dots in Figure 2(b) of the main manuscript and used for further modelling). Figure (a) presents the comparison of the two lines in the linear scales (as measured), while (b) and (c) show the same data and fits, but in logarithmic intensity scales.

The error bars presented throughout the manuscript come from the fitting standard error and the eigenstates energies come solely from the fitting procedure.

IV. MODEL TRANSFORMATION

In ref. [2] with the presentation of the three-mode quantum model, authors show how the 3×3 matrix can be transformed by performing a unitary transformation to block-diagonalize the three-mode system into the two-mode and one-mode subsystems, effectively decoupling the third mode. When the coupling with the mode 0 (the strongly dissipative mode) is weak, such a transformation can be realized in the perturbation method using the Schrieffer-Wolff transformation. The decoupling of the third dissipative mode effectively renormalizes the coupling between modes 1 and 2 and modifies their eigenfrequencies with the additional dissipation (contributing as imaginary parts and guaranteeing the non-negativeness of the dissipation of the model). The authors show how such simplification is valid when $V \ll g_1,\ g_2$, but the coupling to the third state is sufficiently weak (so that the condition for the generator, perturbative and non-perturbative parts of the Hamiltonian is met, see Supplementary Material of ref. [2]). When $V = 0$, effective coupling between the two oscillators g_{12} becomes imaginary when $|E_{1,2} - E_0| \ll |\gamma_{1,2} - \gamma_0|$ and this is realized when mode 0 has large dissipation (γ_0). Hence, to observe attraction, γ_0 should be large such that $|\gamma_{1,2} - \gamma_0|$ is dominated by γ_0, but not so large such that the coupling strength g_{12} is still sizable. Overall it shows a huge importance of the relative ratios of the mode broadenings and their energy differences, on top of the coupling values.

In our case, due to the high importance of the third resonance (the X_Z exciton), and its sizable coupling to both

the photon and the exciton, such a perturbative approximation cannot be made. The 2×2 matrix with the effective imaginary coupling ($g_{12} = V + \frac{1}{2} g_1 g_2 \sum_{i=1,2} \frac{1}{E_i - E_0}$) is not sufficient to describe our system, as the coupling values are sizeable when compared to other energy scales in our system. To visualize it, in Supplementary Figure 3 we present the solution of the transformed 2×2 matrix model (with effective imaginary coupling g_{12}), using the same parameters as extracted from the 3×3 model fitting (see main text). We compare it to the full 3×3 matrix solution, presented with dashed lines. The simplified model (solid) lines clearly deviate from the unsimplified approach (dashed curves) which fits best to our experimental data.

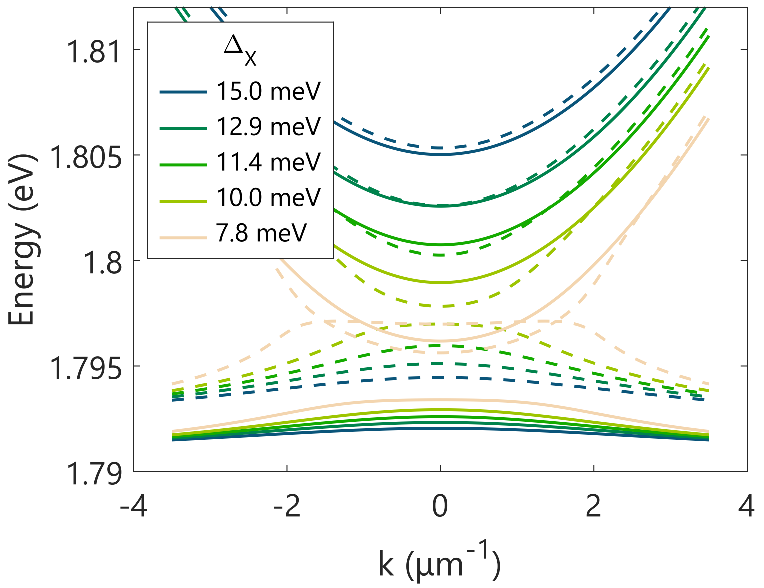

Supplementary Figure 3. Comparison of the 3×3 model solution (dashed lines) with the eigenstates of the model simplified into a 2×2 matrix, by using a Schrieffer-Wolff transformation (described in detail in ref. [2] Supplementary Material, solid lines). All parameters can be found in the main manuscript.

However, even though such a transformation cannot be made in our case, it visualizes the nature of the effect. Anomalous dispersion and band attraction has been previously described by imaginary coupling between two oscillators (see e.g. [3, 4]). In [2] the authors show, how in a real physical system the dissipative coupling can be realized by coupling both oscillators reactively to a third highly dissipative entity. The third-party mode can even be an invisible mode (in our case we identified it as the X_Z-exciton), with high leakage or dissipation. Its effect on the other two resonances is analogous to the imaginary coupling between them - even if the exact transformation into a 2×2 system cannot be made in our case.

V. NON-HERMITICITY AND COUPLING IMPORTANCE

To further visualize the importance of the dissipation of all three modes in the observed attraction effect, in Supplementary Figure 4 we show the solution of the model with varying decay and coupling conditions. Panel (b) shows model curves with no dissipation of the X_Z exciton (the dissipative mode), followed by the model solution with no dissipation of all three resonances (fully real model) in (c). The curves are compared to the model fitted to the experimental data in (a). All three coupling constants V, g_1 and g_2 in (a-c) were fixed, with values extracted from the fitting to the experimental data. One can clearly see, how the non-hermiticity of the system is crucial in the effect observation, with the largest impact of the huge decay of the dissipative mode. Without its contribution, the level attraction cannot be observed and the dispersions become trivial.

Moreover, in panels (d-f) we additionally show the comparison of the solved model with fixed decay constants γ_C, γ_X and γ_0, but varying coupling constants. In panel (d) the modes are fully decoupled, showing the most trivial case of three initial resonances. Panels (e) and (f) show how the lack of coupling between the dissipative mode and the photon (e) or the $X_{X,Y}$ exciton (f) affects the dispersion. One can clearly see how the middle energy branch (solid blue line) in both cases becomes trivial and is hugely redshifted in comparison to the observed data (see panel (a)).

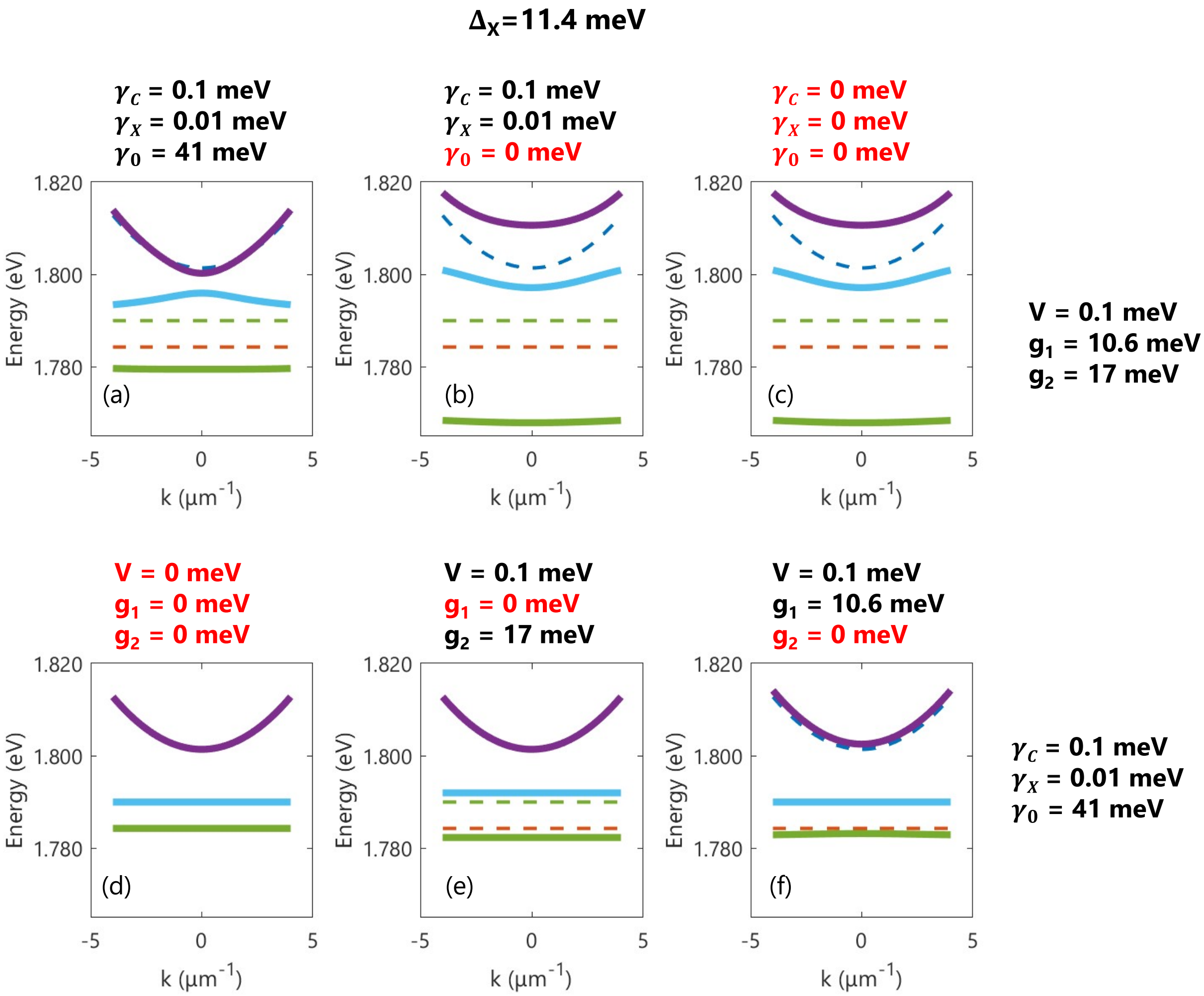

Supplementary Figure 4. Solution of the model with varying decay and coupling conditions, showing the importance of the dissipation and coupling. Solid lines show the dispersions of three eigenmodes, while dashed lines present the initial resonances. Parameters different from the fitted model values (the ones extracted from the experimental data) are marked in red. All curves were calculated at the same detuning between the $X_{X,Y}$ exciton and the photonic mode of 11.4 meV. (a) Model with the fitted parameter values. (b) Model with no dissipation of the lowest energy mode (XZ), $\gamma 0 = 0$. (c) Model with no dissipation of all involved oscillators (fully real model). (d) Model with no coupling between the involved oscillators (fully decoupled modes). (e) Model with no coupling between the X_Z exciton and photon, $g1 = 0$. (f) Model with no coupling between the X_Z and XZ excitons, $g2 = 0$.

VI. HOPFIELD COEFFICIENTS

In addition to our model eigenvalues, we studied also the respective Hopfield coefficients, to gain insight into the contribution of the three involved oscillators in the final modes. By calculating eigenvectors and plotting respective oscillator components we study the dispersion of each contribution. The results at two different experimental detunings ($\Delta_X = 10.0\ meV$, corresponding to a single-maximum dispersion shape of the anomalous branch energy and $\Delta_X = 7.8\ meV$, corresponding to the double-maximum curve) are presented in Supplementary Figure 5.

Left panels (a,e) show the calculated energy dispersions (with three eigenstates labelled as branch 1, branch 2 and branch 3), followed by the Hopfield coefficients for each branch separately in the next columns. In our considerations it's particularly important to look at the mode contributions into the inverted, anomalous branch with the negative effective mass (branch 2, (c) and (g)). Not surprisingly, the excitonic fraction related to the $X_{X,Y}$ exciton is higher than the other two components, due to the energetic closeness between this branch and the initial resonance. However, particularly in the anomalous region around k=0, both other resonances gain importance. Increased contribution of the photon fraction can be considered a typical and an expected behaviour, as it enlarges when the energy proximity

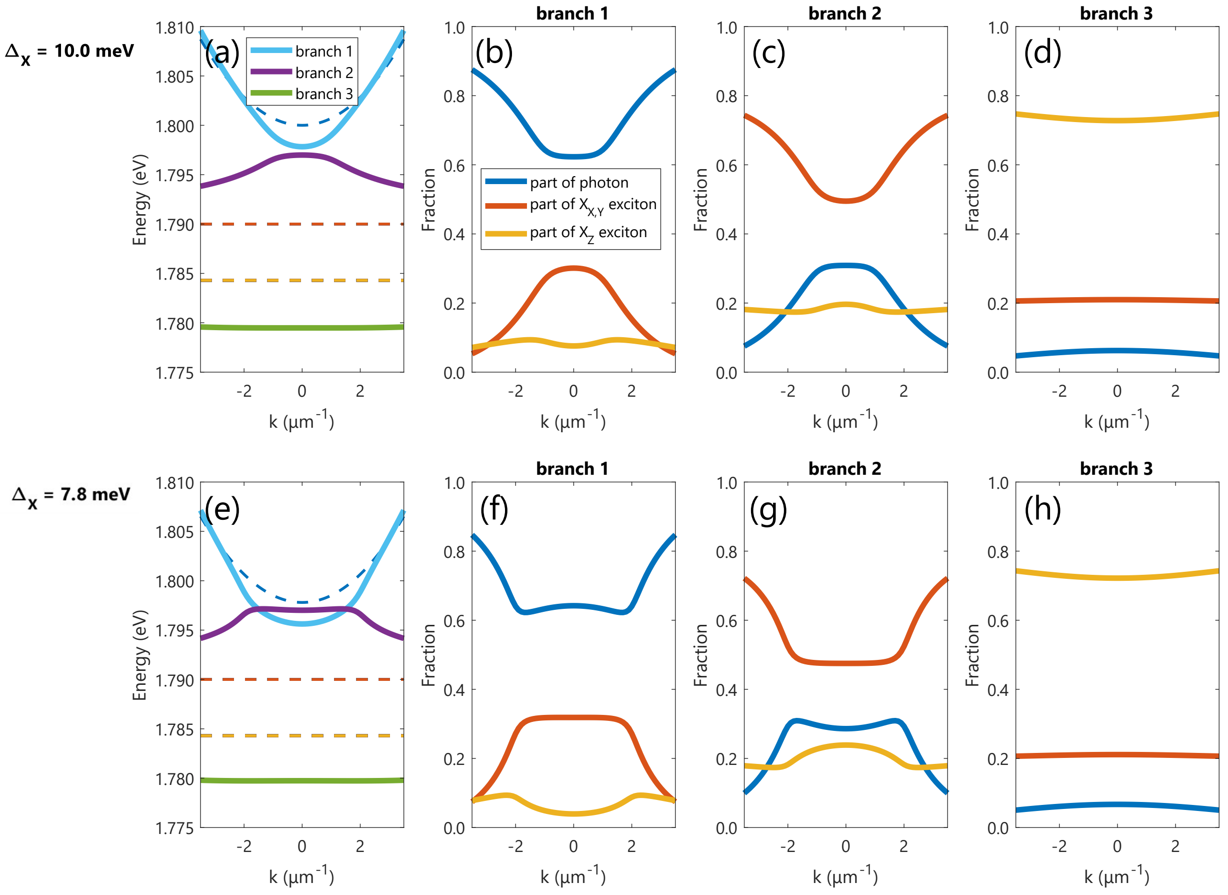

Supplementary Figure 5. Eigenstate dispersions (a,e) and the Hopfield coefficients (b-d,f-h) of each state at two exciton-photon detunings $\Delta_X = 10.0\ meV$ (a-d) and $\Delta_X = 7.8\ meV$ (e-h).

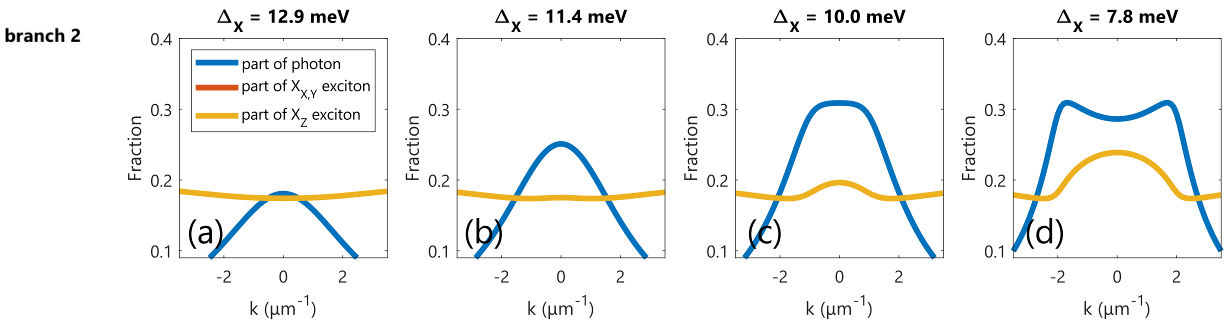

Supplementary Figure 6. Closeup of the Hopfield coefficient dispersions of branch 2 at four exciton-photon detunings. (c) and (d) are the closeup of Supplementary Figure 5 (c) and (g) respectively.

of photon to this final eigenstate is higher. However, the excitonic fraction linked to the X_Z exciton (yellow curve) presents the anomalous dependence near $k = 0$, visible especially at the smaller detuning (bottom row, panels (e-h)). Its value is enhanced in the anomalous region, even though branch 2 diverts from the X_Z resonance, which is a behaviour opposite from a standard Hermitian Hamiltonian. To further visualize this effect we additionally plot the closeup of this region in Supplementary Figure 6, showing only the photonic and the X_Z-excitonic fractions of the branch 2 at four exciton-photon detunings (including the two presented in Supplementary Figure 5).

Increased contribution of the lowest-energy exciton into the anomalous branch in the crucial region, reflecting the inverted shape of the dispersion, pinpoints its interpretation as the dissipative mode. Even though our model cannot be simplified into a 2×2 matrix with imaginary coupling as described in Section IV of this Supplementary Material,

this increased contribution clearly justifies our approach of treating X_Z state as the dissipative mode, crucial to observe level attraction.

VII. EFFECTIVE MASS

Knowing the energy band dispersions one can calculate the particle's effective mass, which is a direct measure of band curvature. A definition of mass, in analogy to solid state systems, can be obtained with the use of a Taylor series expansion of the dispersion [3, 5]: $E(k) \approx E_0 + \frac{\hbar^2 k_0 (k-k_0)}{m_1(k_0)} + \frac{\hbar^2 (k-k_0)^2}{2m_2(k_0)} + \ldots$. The coefficients of each expansion order relate to a new mass parameter, with certain characteristic effects on the dynamics of the particle. In exciton-polariton research the ones most typically defined are:

$$m_1 = \hbar^2 k \left[\partial_k E(k)\right]^{-1} \tag{1}$$

$$m_2 = \hbar^2 \left[\partial_k^2 E(k)\right]^{-1}. \tag{2}$$

The parameter m_1 is related to the classical motion of the wave packet, and determines the group velocity $v_g = \hbar k/m_1$. The parameter m_2 determines the acceleration of the packet when an external force is applied, as well as its rate of diffusion. In case of a purely parabolic dispersion $m_1 = m_2$, but otherwise, m_1 and m_2 can have different signs, be zero, or even become infinite. In typical cases of two strongly coupled oscillators in polariton microcavity the m_1 mass of lower polaritons remains positive for all momenta (while the m_2 effective mass changes sign around the inflection points).

In our case, the m_1 parameter of the anomalous dispersion branch is negative, as it is shown in Supplementary Figure 7. There, in panels (b), (d) and (f) we present the calculated mass m_1 of the inverted branch (branch 2) as a function of the wavevector, with corresponding energy dispersions presented in (a), (c), and (e) respectively. The results are presented at three of the studied detunings Δ_X: 11.4 meV (a-b), 10.0 meV (c-d) and 7.8 meV (e-f). The masses were calculated from the fitted model curves.

One can clearly see, that the group-velocity mass of the anomalous state is negative. With larger detunings $\Delta_X = 11.4$ meV and 10.0 meV the mass $m_1 < 0$ for all momenta (Supp. Fig. 7 (a-d)). At the smaller detuning of 7.8 meV (Supp. Fig. 7 (e-f)) m_1 changes sign at a wavevector $|k^*| \approx 1.34$ μm^{-1} (indicated with the red dotted lines). The closeup of the branch 2 effective mass is also presented in panel (g).

Given the relation $m_1 v_g = \hbar k$, negative mass $m_1 < 0$ means that the particle's velocity and momentum have an opposite sign. Then, the particles will move in the direction opposite to v_g, such that the particles displaced to the positive direction with respect to the excitation spot will have an average velocity towards the same direction [3, 6].

Change of sign of the m_1 parameter at the detuning of 7.8 meV (Supp. Fig. 7 (e-f)) occurs near the inversion peak in the branch dispersion, marked with red dashed lines. However, we also note that this is the detuning at which the discrepancies between the model and the experimental curves are the highest, as discussed in the main text. Regardless, it visualizes the change of the anomalous dispersion of this branch from the single maximum to double-maxima shape and a huge potential of our system to study the exceptional points and related phenomena.

VIII. REFLECTIVITY MEASUREMENTS

In addition to the photoluminescence measurements, we have also measured reflectivity spectra, scanning the same detuning range. We used the same setup as for the photoluminescence studies, but illuminating the sample with the white light source. Three examples of these measurements taken at three different detunings are shown in Supplementary Figure 8(b), (d) and (f), with the corresponding photoluminescence spectra presented in (a), (c) and (e). We also plotted the energies of all three excitons, using dashed lines. In the reflectivity measurements, only the photonic mode can be clearly visible as a dip in the reflectance spectra, characterized by a parabolic dispersion. Neither the anomalous dispersion, nor the bare excitonic levels, are present in the measured spectra.

---

[1] D. Biegańska, M. Pieczarka, K. Ryczko, M. Kubisa, S. Klembt, S. Höfling, C. Schneider, and M. Syperek, Scientific Reports **15**, 10.1038/s41598-025-97221-x (2025).

[2] W. Yu, J. Wang, H. Y. Yuan, and J. Xiao, Phys. Rev. Lett. **123**, 227201 (2019).

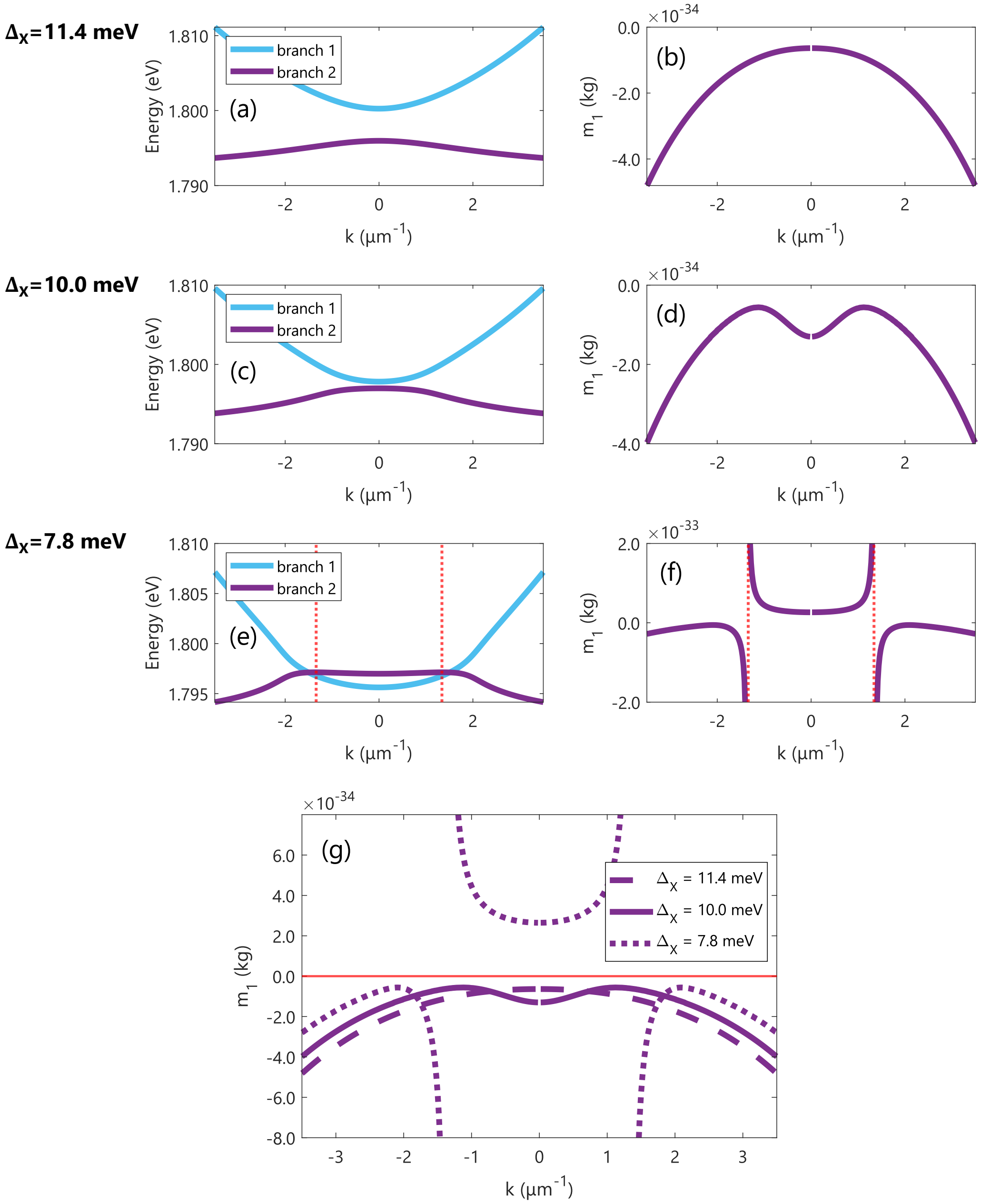

Supplementary Figure 7. Effective mass parameter m_1 wavevector dependence of the investigated anomalous branch (branch 2), at three exciton-photon detunings $\Delta_X = 11.4\ meV$ (a-b), $\Delta_X = 10.0\ meV$ (c-d) and $\Delta_X = 7.8\ meV$ (e-f). Calculated masses are presented in (b), (d) and (f), with corresponding energy dispersions shown in (a), (c) and (e). Red dashed lines in panels (e-f) show the wavevector, at which m_1 parameter of the anomalous branch changes sign. The closeup of these three m_1 wavevector dependencies comparing the three exciton-photon detunings is additionally presented in (g).

[3] M. Wurdack, T. Yun, M. Katzer, A. G. Truscott, A. Knorr, M. Selig, E. A. Ostrovskaya, and E. Estrecho, Nature Communications **14**, 1026 (2023).

[4] O. Bleu, K. Choo, J. Levinsen, and M. M. Parish, Physical Review A **109**, 10.1103/physreva.109.023707 (2024).

[5] D. Colas, F. P. Laussy, and M. J. Davis, Physical Review Letters **121**, 055302 (2018), arXiv:1801.04779.

[6] M. Wurdack, E. Estrecho, S. Todd, T. Yun, M. Pieczarka, S. K. Earl, J. A. Davis, C. Schneider, A. G. Truscott, and E. A. Ostrovskaya, Nature Communications **12**, 10.1038/s41467-021-25656-7 (2021).

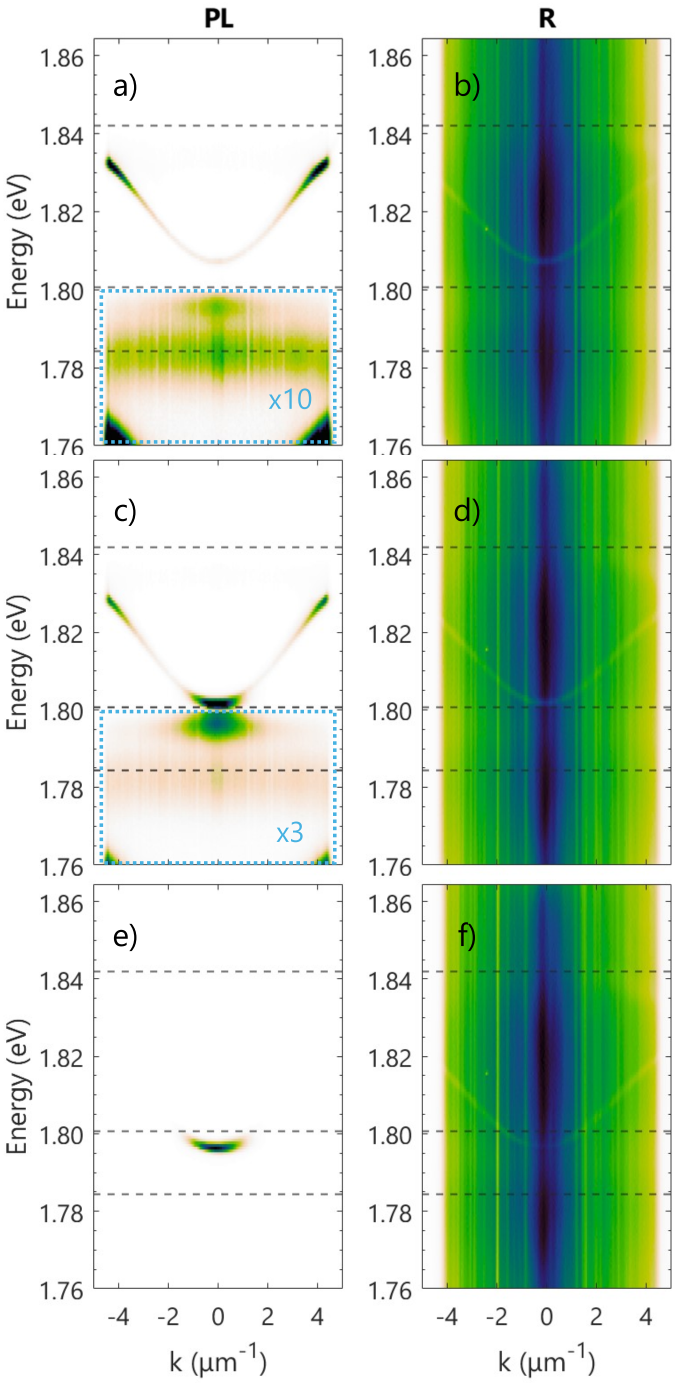

Supplementary Figure 8. Comparison of the photoluminescence (PL, left column) and the reflectivity measurements (R, right column) measured for three exciton-photon detunings in the anomalous regime near $X_{X,Y}$ exciton. For better visibility the intensity within the blue dashed boxes ((a) and (c)) have been multiplied by a factor of 10 (a) or 3 (c). Grey dashed lines indicate the energies of three excitons present within the system. Only the photonic mode is visible in the reflection measurements.
